# Video-Based Detection of Dairy Cow Hoof-Slipping Behaviour Using Improved DeepLabCut and NeuFlow v2

**DOI:** 10.3390/ani16132103

**Published:** 2026-07-07

**Authors:** Yue Nian, Kaixuan Zhao, Jiangtao Ji, Yinan Chen, Ruihong Zhang

**Affiliations:** 1 College of Agricultural Equipment Engineering, Henan University of Science and Technology, Luoyang 471023, China; y.nian@stu.haust.edu.cn (Y.N.); chenyn@haust.edu.cn (Y.C.); hkdzrh@163.com (R.Z.); 2 Science & Technology Innovation Center for Completed Set Equipment, Longmen Laboratory, Luoyang 471023, China; jjtao@haust.edu.cn

**Keywords:** dairy cow slipping, animal welfare, CA attention mechanism, NeuFlow v2 algorithm, random forest algorithm

## Abstract

Hoof slipping in dairy cows is a subtle, transient hoof motion event distinct from lameness or falling, with short duration, limited displacement, and close resemblance to normal gait, making automated detection particularly challenging; relevant methods remain scarce. This study proposes a cascaded detection framework based on improved DeepLabCut and NeuFlow v2 for automated hoof-slipping detection and distance estimation in Holstein dairy cows. The four-stage framework covers hoof key point localization, pixel-level optical flow fusion, motion parameter curve feature extraction, and Random Forest classification. The framework was developed on Dataset 1, which contained 115 single-cow side-view videos. Of these, 31 contained slipping events and 84 were normal walking. It was further assessed on a smaller second-farm dataset of 17 single-cow videos (Dataset 2). ResNet-50 with a Coordinate Attention mechanism was adopted as the backbone, reducing mean four-hoof localization RMSE to 2.80 pixels across five independent training runs, showing a 15.2% improvement over the baseline, and outperforming YOLOv8s-Pose. NeuFlow v2 was applied to extract the localized optical flow from hoof regions, yielding velocity and directional curves from which slipping features were derived. The Random Forest classifier achieved an accuracy of 98.9%, precision of 93.3%, recall of 90.3%, F1 score of 91.8%, and AUC of 0.995, outperforming MViT, SlowFast, and STME. The slipping distance estimation RMSE was 1.22 pixels. With the localisation model retrained on new farm frames, the method reached comparable performance on the second farm, suggesting preliminary cross-farm generalisability that warrants larger-scale validation. The proposed framework provides a non-invasive basis for early hoof-health monitoring and welfare-oriented farm management.

## Simple Summary

Hoof slipping in dairy cows can signal hoof disease or dangerous floor conditions, but it is extremely difficult to detect automatically because it is very brief and closely resembles normal walking. This study developed a system that uses video recordings to automatically detect and measure hoof slipping. The system analyzes each video frame to locate the hooves, tracks their movement, and determines whether slipping has occurred. The system achieved an overall accuracy of 98.9% and successfully identified 90.3% of all actual slipping events. Initial testing on a second farm showed promising performance across different environments. This technology could help farmers detect hoof-health problems earlier and improve animal welfare on dairy farms.

## 1. Introduction

Dairy cows play a crucial role in animal husbandry, and their productivity and economic contributions are closely linked to the development of the industry [1]. Contemporary animal welfare science has developed the Five Domains Model, which evaluates animal welfare across five dimensions—nutrition, environment, health, behavioural interactions, and mental state [2]; hoof health is central to meeting these welfare standards, as compromised hooves not only directly restrict locomotion but are also a well-established source of chronic pain that can lead to neuroinflammatory changes in the central nervous system of dairy cattle. As the degree of intensification of dairy farms increases, reduced activity space and harder ground surfaces have elevated the incidence of hoof and limb diseases [3]; long-term standing on cement floors inflicts mechanical trauma on the hooves, while high humidity and heat stress during summer months soften the keratinous tissue, further increasing the risk of hoof-related ailments [4]. Hoof diseases, including interdigital dermatitis, can cause dairy cows to slip or stumble while walking [5,6], while also impairing appetite and locomotive ability, resulting in decreased feed intake and reduced milk production [7]. From an animal welfare perspective, hoof pain is one of the most significant sources of suffering in intensive dairy systems; affected cows exhibit behavioural indicators of compromised welfare, including prolonged lying time, reluctance to walk, and altered weight-bearing postures [8], and given that hind limbs bear most of their weight, the culling rate due to hoof disease is exceedingly high [9]. Ground hardness, flatness, and humidity, as factors of an inappropriate breeding environment, can also induce hoof slipping, compromising animal welfare and inflicting significant economic losses on dairy farmers [10]. In this study, hoof slipping is therefore treated not as a sign of a single cause but as a non-specific behavioural marker of compromised locomotor stability, which may arise from underlying hoof disorders, from hazardous flooring conditions, or from a combination of the two. Therefore, timely detection of hoof slipping serves dual purposes: triggering early welfare interventions and enabling the objective assessment of housing conditions to guide welfare-oriented farm management.

In recent years, numerous scholars have investigated the movement patterns of cows’ heads, necks, backs, and hooves to elucidate the characteristics of bovine lameness [11], with machine vision technology being particularly suitable for such monitoring due to its noncontact and stress-free nature [12,13]. Representative studies include the following: Zhao et al. [14], who located key points on a cow’s body and extracted head–hind-hoof linkage features for early lameness detection; Li et al. [15], who utilized an enhanced SOLOv2 network combined with Hungarian and Canny algorithms for multidimensional lameness feature extraction; and Barney et al. [16], who enhanced Mask-RCNN with key point outputs and CatBoost classification for automated lameness detection. Additional studies have also explored cow lameness detection from other perspectives [17,18]. These methods generally locate body key points and track their trajectories to identify abnormal movement patterns, demanding high precision in key point localization; however, achieving near-perfect positioning accuracy remains challenging.

As demonstrated by the aforementioned studies, lameness detection is essentially a form of global gait abnormality detection. It characterizes a persistent locomotor disorder by integrating whole-body kinematic cues—including head–hoof coordination patterns, dorsal arching features, and limb-swing trajectories—across multiple complete gait cycles. Spatially, lameness-related features are distributed across the entire body of the cow; temporally, they persist continuously and recur with regularity at the scale of complete gait cycles. This stands in fundamental contrast to hoof slipping. As a transient, sporadic, and subtle behavioural event, hoof slipping produces an anomalous signal that is confined to a single hoof, lasts only a few frames, and is readily obscured by the dominant normal motion of the rest of the body. This pronounced difference from lameness in both spatial and temporal scales indicates that the global gait modelling strategy employed in lameness detection cannot be directly transferred to hoof-slipping detection. Consequently, detecting hoof slipping requires an approach that first anchors the analysis to the precise hoof region and subsequently performs pixel-level transient motion analysis within that region to extract the anomalous signal. To the best of our knowledge, pixel-level transient motion analysis targeting localized hoof events has not yet been investigated in the domain of dairy cow monitoring. This methodological gap constitutes the direct motivation for the cascaded framework proposed in this study.

In the domain of slip detection, visual detection research targeting human falls has reached a relatively mature stage and has provided important methodological references for abnormal animal behaviour detection. Núñez–Marcos et al. [19] proposed a fall detection system combining optical flow with convolutional neural networks; Chhetri et al. [20] further developed a dynamic optical flow enhancement technique using rank pooling to compress optical flow videos into a single image, improving detection accuracy under dynamic lighting; and Fei et al. [21] constructed a dual-stream network, Flow-Pose Net, by integrating optical flow with skeletal pose to enhance detection robustness. However, these methods all target drastic changes in overall human posture, making them difficult to directly transfer to cow hoof-slipping detection, which involves localized and subtle movements. Unlike human falls, hoof-slipping is an instantaneous abnormal movement at the hoof, characterized by extremely short duration, limited displacement, and susceptibility to confusion with normal gait movements, thereby imposing higher demands on feature extraction granularity. Furthermore, end-to-end action recognition methods are ill-suited for this task, as they extract sustained, holistic motion patterns whose global spatiotemporal features are dominated by the routine movements of other body parts, causing localized, transient abnormal signals to be diluted. Effective hoof slipping detection therefore requires an approach that spatially anchors the hoof position while capturing subtle motion changes at the temporal level. To the best of our knowledge, automated detection methods specifically designed for hoof slipping in dairy cows remain scarce.

To address this challenge, this study proposes a cascaded detection framework based on improved DeepLabCut and NeuFlow v2 for automated hoof-slipping detection and slipping distance estimation in dairy cows. The framework integrates hoof key point localization with a Coordinate Attention-enhanced ResNet-50 backbone, pixel-level optical flow fusion via NeuFlow v2, motion feature extraction, and Random Forest classification, forming a complete pipeline from hoof localization to slipping identification. This work provides a non-invasive basis for early hoof-health monitoring and welfare-oriented farm management, advancing precision livestock farming in support of improved animal welfare.

## 2. Materials and Methods

### 2.1. Experimental Datasets

This study utilized datasets collected from two independent farms, which differed substantially in geographical location, data collection period, floor material, recording equipment, and capture parameters, providing a basis for method validation and generalizability assessment.

**Figure 1:**
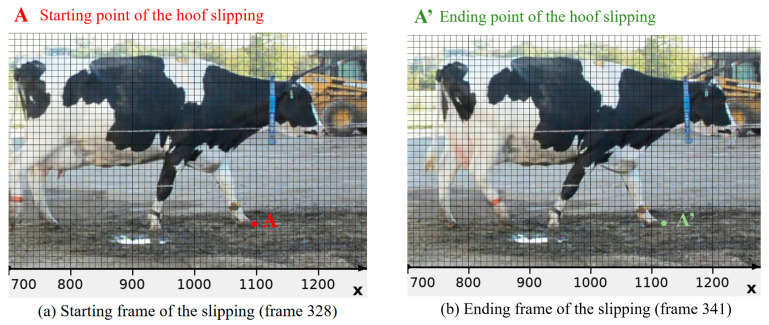
Demonstration of forward slipping from a cow’s left front hoof.

Dataset 1 was collected from August to November 2016 at the University of Kentucky Research Dairy Farm in the United States. The 52 subjects were lactating Holstein cows. Data were collected under two natural lighting conditions, sunny and cloudy days, with collection times covering all periods throughout the day. A Nikon D5200 camera (Nikon, Tokyo, Japan) equipped with a 35 mm lens with a sensitivity of 400 ISO was used and set to automatic exposure and autofocus modes. The camera was mounted on a tripod 1.5 m above the ground and 3.5 m from the channel, with the shooting direction perpendicular to the cow walking channel. To ensure that the video captured two complete movement cycles of the cow, the camera position was adjusted to achieve a video field width of approximately 6.8 m, and a reference rope was horizontally set up in the centre of the shooting channel for on-site measurement to confirm this distance. The frame rate was 59.94 fps, and the bit rate was 20 Mbps. The resolution of each frame was 1280 pixels (horizontal) × 720 pixels (vertical). A total of 115 videos was collected in this study, each lasting approximately 10–20 s and featuring a single cow. As the herd comprised 52 cows and recordings were made at different times, individual cows could appear in more than one video. Slipping is a sporadic event whose occurrence depends on the instantaneous walking conditions at the time of recording, such as floor wetness and landing posture, rather than on a fixed attribute of the cow. Recordings of the same cow made at different times were therefore regarded as approximately independent observations at the event level. Of these, 31 were manually identified as containing hoof-slipping events, and the remaining 84 consisted of normal walking footage. Figure 1 shows an example of a cow’s left front hoof slipping forward.

Dataset 2 was collected in February 2026 at Shengsheng Farm in Luoyang, Henan Province, China, with lactating Holstein dairy cows as the subjects. The floor of the cow passage was covered with hard rubber matting. An Intel RealSense D455 (Intel Corporation, Santa Clara, CA, USA) depth camera was used for recording, mounted on a tripod at a height of 1.27 m above the ground and 3 m from the cow walking passage, with the shooting direction perpendicular to the passage. The video resolution was 1280 × 720 pixels at a frame rate of 30 fps. A total of 17 single-cow side-view walking videos and 15 multi-cow parallel walking videos was collected, each approximately 10 to 20 s in duration. Of the single-cow side-view walking videos, six were manually identified as containing hoof-slipping events, and the remaining 11 consisted of normal walking footage.

Unless otherwise specified, all method descriptions, experiments, and analyses in this study are based on Dataset 1. Dataset 2 was used exclusively for cross-farm generalization validation and the exploration of multi-cow settings. As the proposed pipeline targets the single-cow side-view scenario, the cross-farm experiment used only videos of this type. The multi-cow videos served not as training data but as a preliminary exploration of a potential multi-cow extension (Section 4.4). The generalizability of this study is therefore limited to this scenario.

### 2.2. Overall Workflow for Slipping Feature Extraction

**Figure 2:**
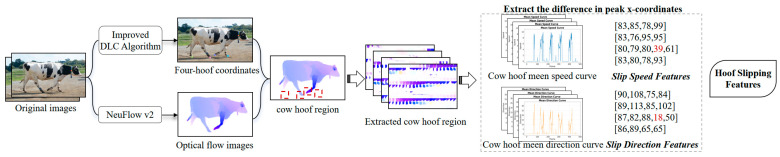
Slip feature extraction flowchart.

In this study, the enhanced DeepLabCut key point positioning model was employed to locate the key points of the cow’s four hooves and obtain their coordinates in the side-view images of the cow walking. Concurrently, NeuFlow v2 was used to infer the optical flow information from the side-view videos of the cow walking, thereby generating optical flow maps. The side-view walking images of the cow and optical flow images were matched frame by frame to identify the regions corresponding to the four hooves in the optical flow images. Centred on the coordinates of the cow’s four hooves, a 20 × 20 pixel region was extracted from the optical flow image for each hoof. Subsequently, white pixels within the hoof regions of the optical flow images were removed to isolate the optical flow information specific to the cow’s hooves. Hoof motion speed and direction curves were derived from the denoised optical flow data. Peak detection was performed on the extracted hoof speed and direction curves. The differences between the horizontal coordinates of adjacent peaks in the speed curve were quantified to form slipping speed features, whereas those in the direction curve were used to construct directional slipping features. The complete workflow is illustrated in Figure 2.

### 2.3. Improved DeepLabCut for Hoof Key Point Localization

#### 2.3.1. Improved DeepLabCut Model

DeepLabCut is a widely used toolbox for animal pose estimation that leverages transfer learning and data augmentation techniques to achieve satisfactory training outcomes even with limited training data [22].

**Figure 3:**
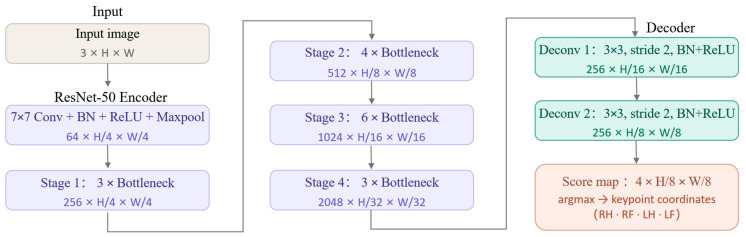
Network architecture of DeepLabCut.

To identify the optimal backbone for detecting hoof key points, this study evaluated 10 models within the DeepLabCut framework: ResNet-50, ResNet-101, ResNet-152 [23], MobileNet-V2-0.35, MobileNet-V2-0.5, MobileNet-V2-0.75, MobileNet-V2-1.0 [24], EfficientNet-b0, EfficientNet-b3, and EfficientNet-b6 [25]. Based on the training outcomes, ResNet-50 was selected for further enhancement. As shown in Figure 3, the DeepLabCut framework consists of a ResNet-50 encoder that progressively reduces spatial resolution to H/32 × W/32, and a decoder with two transposed convolutional modules that restore resolution to H/8 × W/8. The final output is a four-channel score map corresponding to the four-hoof key points, from which precise coordinates are extracted using the argmax operation.

**Figure 4:**
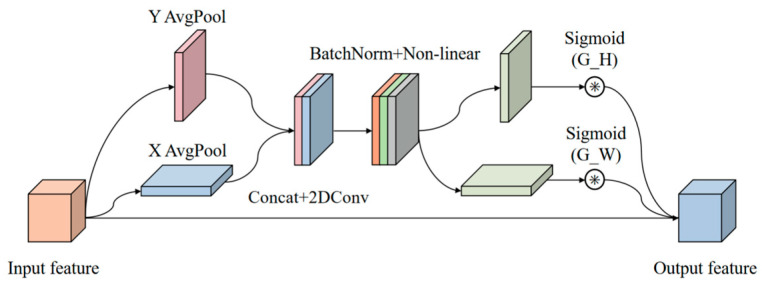
Structural diagram of CA.

To enhance the generalization ability and robustness of the model, a Coordinate Attention (CA) mechanism was introduced to optimize the ResNet-50 network architecture. Specifically designed for mobile networks, this lightweight attention mechanism embeds positional information into channel attention to achieve collaborative modelling of spatial coordinates and channel features. CA [26] encodes input feature maps along the horizontal and vertical directions through two parallel one-dimensional pooling operations, thereby simultaneously capturing cross-channel feature dependencies and precise directional positional information. Its structure is illustrated in Figure 4. Four-hoof key point localization in dairy cows is essentially a precise coordinate regression task targeting specific positions in the image, which places high demands on the preservation and propagation of spatial positional information throughout the feature maps. Moreover, the four key points exhibit clearly defined spatial distribution relationships along both the horizontal and vertical directions, requiring the model to maintain sensitivity to spatial coordinates throughout the feature extraction process. By performing feature aggregation separately along the horizontal and vertical directions, CA establishes bidirectional positional awareness, allowing it to encode spatial coordinate information into the feature representation while simultaneously recalibrating channel importance. This structural property of CA aligns closely with the demands of the key point localization task in this study.

**Figure 5:**
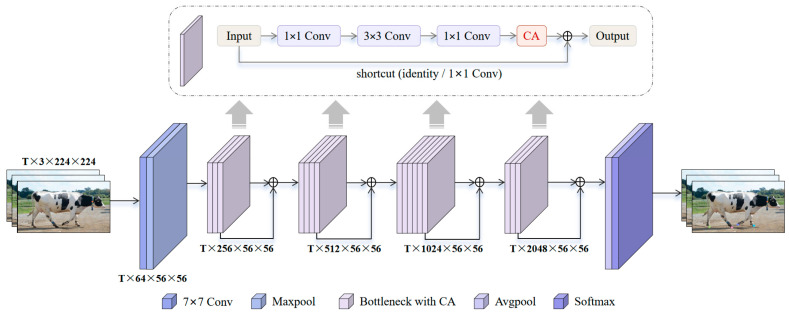
Overall architecture of the improved ResNet50 network.

To address the optimization requirements of feature extraction in the ResNet-50 network under complex conditions, this study embedded the CA module at the end of the residual branch of each Bottleneck block, prior to the element-wise addition with identity mapping (shortcut). Crucially, this leaves the identity shortcut unobstructed, preserving the direct gradient pathway that is foundational to the trainability of deep residual networks. The CA recalibration is thereby confined to the residual branch, acting on the incremental features learned within each block rather than on the fused output; inserting CA after the element-wise addition would instead operate on the combined signal and interrupt this gradient highway. This design enables attention-based recalibration to act consistently at every depth level of the backbone network, allowing feature calibration to operate continuously from low-level texture features to high-level semantic representations across all stages, thereby ensuring that spatial positional information is effectively preserved and propagated throughout the entire feature extraction process. The complete architecture of the improved network is shown in Figure 5. To validate the rationale behind the selection of the CA mechanism, ablation experiments encompassing SE [27], ECA [28], CBAM [29], and CA were conducted for comparative verification, as discussed in the Results section.

#### 2.3.2. Key Point Annotation and Evaluation Metrics

**Figure 6:**
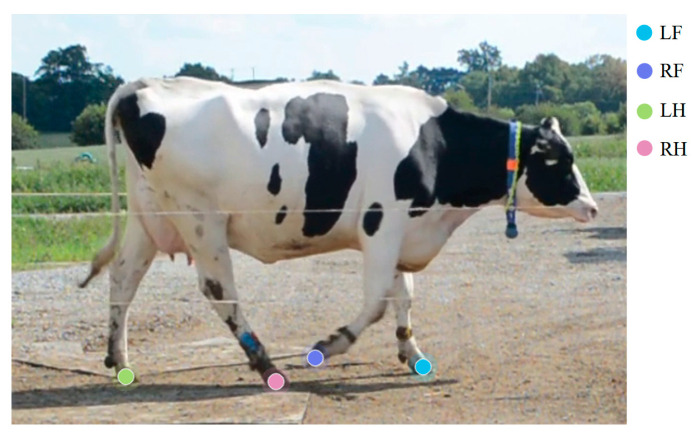
Key point annotation example.

The DeepLabCut annotation tool was employed to label the key points of the cow’s four hooves, as depicted in Figure 6. During the annotation process, the left front hoof (LF), right front hoof (RF), left hind hoof (LH), and right hind hoof (RH) were individually labelled.

The root mean square error (RMSE) was selected as the evaluation metric for assessing the quality of key point predictions for quadrupedal hooves. The RMSE quantifies the deviation between the predicted and ground truth values with the following mathematical formulation:

(1) $$X_{\mathrm{RMSE}}=\sqrt{\frac{1}{N}\sum_{i=1}^{N}{\left(X_{\mathrm{obs},i}-X_{\mathrm{model},i}\right)}^{2}}$$

where N denotes the number of observations; $X_{obs}$ the observed value for the i-th sample; and $X_{model}$ the ground truth value.

### 2.4. Optical Flow Estimation Calculation

#### 2.4.1. Selection of Optical Flow Models

This study conducted a systematic performance evaluation of five representative optical flow algorithms: NeuFlow v2 [30], SEA-RAFT [31], FlowFormer++ [32], GMFlow [33], and FlowNet2.0 [34]. NeuFlow v2 adopts a global-to-local hierarchical feature matching strategy for efficient real-time inference. SEA-RAFT employs a recurrent iterative optimization framework with spatially enhanced attention. FlowFormer++ leverages the Transformer architecture to capture global contextual information, achieving high accuracy at the cost of inference latency. GMFlow reformulates optical flow estimation as a single-pass global matching problem. FlowNet2.0, as an early deep learning-based method, integrates multiple subnetworks for pixel-level prediction. This evaluation aims to provide a reliable basis for method selection when transferring to real dairy farm environments.

#### 2.4.2. Evaluation Setup for Optical Flow Algorithms

This study employed the KITTI Flow 2015 [35] dataset to evaluate the performance of each optical flow algorithm. This dataset was collected from real outdoor environments and compared to synthetic rendered datasets; it is more closely aligned with the video data from real dairy farm environments in terms of image capture modality, illumination conditions, and scene complexity. Both the dataset and video data were captured under natural lighting conditions, featuring the genuine motion of dynamic subjects and non-uniform background textures. Therefore, this dataset can more objectively reflect the generalization capability of each algorithm under real-world shooting conditions, providing a more informative and reliable evaluation reference for subsequent optical flow extraction in real farm scenarios.

This study employed the average endpoint error (AEE) and the percentage of optical flow outliers (Fl) as the primary performance metrics, and evaluations were conducted separately for occluded regions (occ) and non-occluded regions (noc), while inference time was also recorded to assess the practical deployment feasibility of each algorithm. The average endpoint error quantifies the spatial discrepancy between the predicted and ground truth optical flow vectors. The endpoint error (EPE) is defined as the Euclidean distance between the endpoints of the predicted and true flow vectors, with the AEE representing the mean EPE across all pixels. The mathematical formulation is expressed as follows:

(2) $$EPE=\sqrt{{\left(u_{E}-u_{G}\right)}^{2}+{\left(v_{E}-v_{G}\right)}^{2}}$$

(3) $$AEE=\frac{1}{N}\sqrt{{\left(u_{E}-u_{G}\right)}^{2}+{\left(v_{E}-v_{G}\right)}^{2}}$$

The percentage of optical flow outliers (Fl) is defined as the proportion of pixels whose endpoint error simultaneously exceeds three pixels and 5% of the ground truth magnitude relative to the total number of pixels. The mathematical formulation is expressed as follows:

(4) $$Fl=\frac{1}{N}\underset{x}{\sum }1[EPE\left(x\right)>3px\wedge \frac{EPE\left(x\right)}{\left|f^{⁕}\left(x\right)\right|}>0.05]$$

In Equations (2) and (3), $u_{E}$ and $v_{E}$ denote the horizontal and vertical components of the predicted flow, whereas $u_{G}$ and $v_{G}$ represent the corresponding ground truth components. $f^{⁕}\left(x\right)$ denotes the ground truth optical flow vector at pixel x, and N is the total number of valid pixels.

### 2.5. Slipping Feature and Distance Detection

#### 2.5.1. Hoof Motion Speed and Direction Calculation

**Figure 7:**
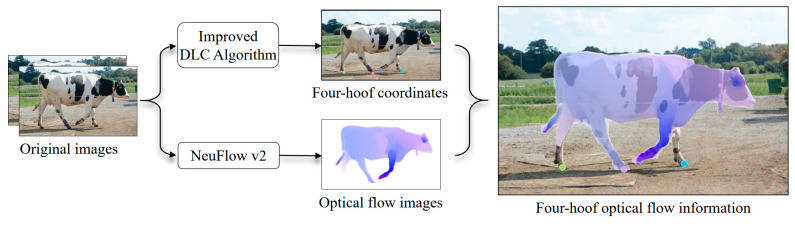
Key point fusion by NeuFlow v2 for extraction of optical flow of hooves.

The enhanced DeepLabCut model was employed to predict hoof key points in preprocessed lateral-view videos, yielding four-hoof coordinates for each frame. Concurrently, NeuFlow v2 was utilized to extract optical flow information from the same videos. The predicted key point coordinates were matched with the optical flow images on a frame-by-frame basis, accurately mapping each hoof position onto the corresponding optical flow image. The overall process is illustrated in Figure 7.

To isolate hoof-specific optical flow information, square regions of 20 × 20 pixels were extracted centred on each hoof key point. The region size reflects a trade-off between coverage and interference. A region that is too small is sensitive to key point localisation error and may miss part of the hoof motion. A region that is too large, by contrast, admits background flow and interference from the adjacent hoof when the two hooves overlap. A 20 × 20 region covers the hoof motion range while suppressing such interference. This region size was determined through ablation experiments comparing 15 × 15, 20 × 20, and 30 × 30 pixels, with 20 × 20 identified as optimal. The extracted regions were categorized into LF (left fore), LH (left hind), RF (right fore), and RH (right hind). White pixels within the hoof regions were removed through transparency processing to mitigate interference in subsequent speed and direction extraction.

The speed and direction were extracted for each optical flow image, with the horizontal (u) and vertical (v) optical flow components of each pixel within the image being specifically identified. The optical flow speed of each pixel is calculated as follows:

(5) $$s=\sqrt{u^{2}+v^{2}}$$

The optical flow direction a of each pixel point is calculated as follows:

(6) $$a=\frac{180\times arctan2\left(v,u\right)}{\pi }$$

We assume that the number of pixels in each image is n and the optical flow speed $\bar{s}$ and optical flow direction $\bar{a}$ of each image are as follows:

(7) $$\bar{s}=\frac{s_{1}+s_{2}+s_{3}+\ldots +s_{n}}{n}$$

(8) $$\bar{a}=\frac{a_{1}+a_{2}+a_{3}+\ldots +a_{n}}{n}$$

Then, speed and direction curves can be obtained for each hoof across all optical flow frames.

#### 2.5.2. Calculation of Slipping Features

The slipping feature of a dairy cow is defined as an array comprising the differences between the horizontal coordinates of the peak points of the hoof speed and direction curves. Each cow’s four hooves correspond to four speed curves and four direction curves, meaning that one cow can yield four sets of slipping speed features and four sets of slipping direction features.

**Figure 8:**
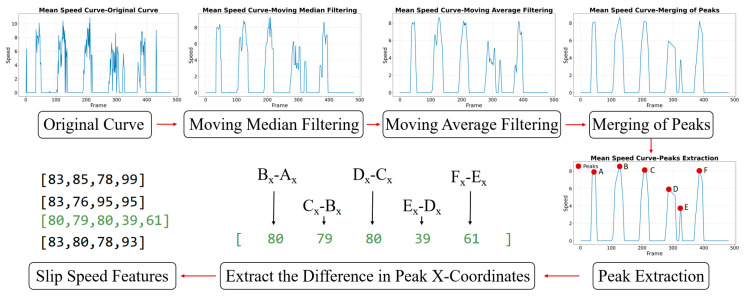
Process of extracting slipping feature parameters.

**Figure 9:**
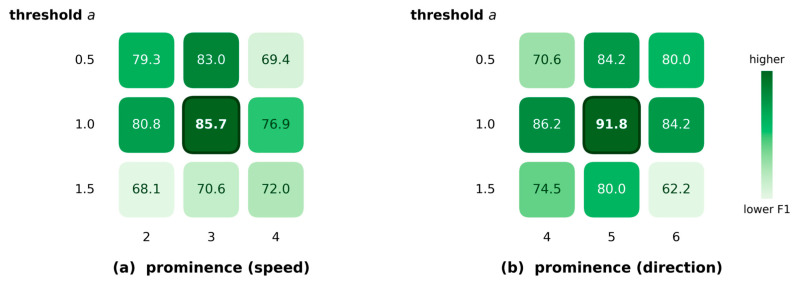
Grid search results for peak-merging hyperparameters. Cross-validated F1 (%) for (**a**) the speed curve and (**b**) the direction curve. The outlined cell marks the selected setting.

The process of extracting the slipping features of cow hooves is illustrated in Figure 8. After the speed and direction curves for the cow’s four hooves are obtained, the initial step involves filtering these curves. A moving median filter was applied to the curves, with a sliding window size set to five. A moving average filter was subsequently used to smooth the curves, and a sliding window size of five was used. To address the multiple peaks in the curves, peaks were merged with the following logic: A threshold a is established. If the values between peak points consistently exceed a, the two peak points are connected and merged; if the values between peak points are less than a, they remain unchanged. In this study, threshold a was set to one. Peaks were detected using the find_peaks function from the SciPy signal library. A candidate peak is a local maximum, and its prominence, defined as the height of the peak relative to the lowest contour line that does not enclose a higher peak, was used to discard minor peaks below the prominence threshold. A higher prominence value indicates a more pronounced fluctuation in the peak relative to adjacent data, thereby highlighting more significant peaks. In this study, the prominence features were set to three for the speed curve and five for the direction curve. After peak merging, the horizontal coordinates of the peak points were extracted. The two hyperparameters, the merging threshold a and the prominence, were determined by grid search. The threshold a was varied over {0.5, 1.0, 1.5}; the prominence was varied over {2, 3, 4} for the speed curve and {4, 5, 6} for the direction curve. Each combination was evaluated by the cross-validated F1 score of the downstream slipping classifier. As shown in Figure 9, the highest F1 was obtained at a = 1.0 with a prominence of three for the speed curve and at a = 1.0 with a prominence of five for the direction curve.

#### 2.5.3. Slip Detection with Random Forest

Analysis of the extracted slipping features of cow hooves revealed that the value for these features is abnormally low when a cow experiences hoof-slipping. In this study, supervised Random Forest (RF) [36] and XGBoost [37] classifiers were employed to identify and classify hoof slipping behaviour based on the extracted hoof motion features. RF aggregates predictions from multiple independent decision trees to reduce variance, while XGBoost sequentially fits residuals through gradient boosted trees with regularization.

The extracted slipping feature parameters were used to develop two classification models: one based on speed magnitude curves and the other on direction curves. Because hoof slipping is a brief event confined to a single limb, only the affected hoof of a slipping cow exhibits an anomalous signal, while its other three hooves maintain the normal gait pattern and therefore constitute valid normal samples. This localized nature justifies treating each hoof as an independent unit at the sample level. A total of 115 side-view walking videos was collected, from which 460 sets of slipping features were extracted. All four hooves of normally walking cows contributed 336 normal samples, the affected hooves of slipping cows contributed 31 abnormal samples, and the remaining three hooves of slipping cows contributed 93 normal samples, yielding a total of 429 normal samples and 31 abnormal samples. This sample distribution is markedly imbalanced. To mitigate classifier bias toward the majority (normal) class and preserve sensitivity to the minority (slipping) class, the class_weight parameter of the Random Forest was set to “balanced”. This setting assigns each class a weight inversely proportional to its frequency, allowing the under-represented slipping class to contribute proportionally more to the training objective. Individual-level factors shared among the four hooves of a cow, such as gait rhythm, illumination, and camera viewpoint, could leak between training and evaluation. To prevent this, all four hooves recorded in the same video were assigned to the same fold, and grouped five-fold cross-validation was applied. As overall accuracy can be misleading under class imbalance, precision, recall, F1 score, and AUC for the slipping class were adopted as the primary performance metrics. To verify the stability of the main classifier in this study, the grouped five-fold cross-validation was repeated 20 times with different random seeds. For each metric, the mean, the standard deviation and the 95% empirical interval across all folds were recorded. A confusion matrix was aggregated from the out-of-fold predictions of a representative run. The precision and recall of each fold were also reported to check whether both remain consistent across folds.

#### 2.5.4. Slip Distance Detection

**Figure 10:**
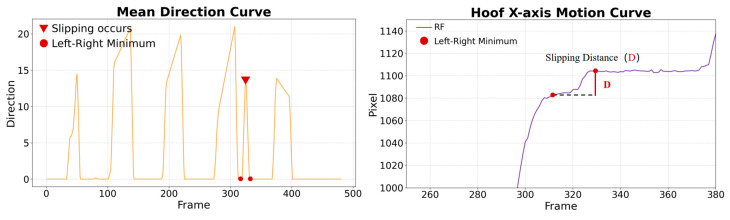
Slipping distance detection diagram.

Since the direction feature demonstrated higher classification accuracy, it was used for slipping distance detection. The detection process is shown in Figure 10. When the Random Forest identifies an abnormally low minimum value in a hoof’s direction-feature array, that hoof is marked as the slipping hoof. The abnormal minimum value corresponds to two adjacent peak points in the direction curve, of which the smaller one is identified as the slipping peak. The lowest points on both sides of the slipping peak are then located, and their horizontal coordinates are mapped to the corresponding positions on the hoof’s X-axis trajectory curve. The difference in the X coordinates (horizontal hoof positions) of these corresponding points yields the slipping distance D. RMSE was selected to evaluate the accuracy of slipping distance estimation. During data acquisition, a reference rope was stretched horizontally across the centre of the shooting channel. On-site measurement confirmed that the rope spanned 6.8 m across the field of view, corresponding to 1280 pixels, yielding a pixel-to-physical scale factor of 5.31 mm/px. Since hoof slipping predominantly occurs in the horizontal direction, this scale factor was used to convert the slip distance from pixels to actual physical distance.

### 2.6. Experimental Settings

All experiments in this study were conducted on the same hardware platform, comprising an NVIDIA GeForce RTX 4070 GPU (NVIDIA Corporation, Santa Clara, CA, USA) with 24 GB of memory and an Intel^®^ Core™ i7-12700KF CPU (Intel Corporation, Santa Clara, CA, USA), ensuring that comparisons across different models were performed under identical computational conditions.

For the key point localization experiments, 1000 annotated frames were uniformly sampled at even time intervals within each of the 84 normal walking videos in Dataset 1 and divided into training and test sets at a ratio of 8:2. Because the frames were sampled uniformly in time, the sampled frames within each video are temporally well-separated rather than adjacent, so near-duplicate frames do not appear across the training and test sets. The multi-animal imgaug data augmentation strategy built into DeepLabCut was applied, incorporating transformations including rotation, scaling, flipping, cropping, brightness adjustment, and contrast adjustment. Model training used the Adam optimizer with an initial learning rate of 0.0005, which was reduced to 0.0001 at iteration 7500 and further to 0.00005 at iteration 12,000. The total number of training iterations was 60,000, and the key point prediction probability threshold was set to 0.6. The model was implemented in Python 3.7 (Python Software Foundation, Wilmington, DE, USA), with CUDA 11.3 and cuDNN 8.2.0 (NVIDIA Corporation, Santa Clara, CA, USA) as the GPU acceleration framework. In the backbone network comparison experiments, all ten candidate networks were trained under the same configuration and hardware conditions were described above. In the attention mechanism ablation experiments, all four attention modules were compared at a unified integration position under identical training parameters and hardware conditions, ensuring a fair evaluation.

For comparison, YOLOv8s-Pose (Ultralytics, Los Angeles, CA, USA) was trained on the same annotated data and evaluated on the same test set as the CA-enhanced ResNet-50. An identical RMSE definition was used to ensure a fair comparison. The four-hoof key points were converted to the YOLO pose format. As the cow hooves are confined to the lower part of the side-view image, the lower half of each frame was used as the bounding box for pose estimation, so that the evaluation isolated key point regression performance from object detection. The model was fine-tuned from the official yolov8s-pose.pt pretrained weights. An input resolution of 960 was used, with rectangular training enabled in order to preserve the original aspect ratio. Training used the default SGD optimizer of the YOLOv8 framework with an initial learning rate of 0.01, a batch size of eight, and 300 epochs, on the same hardware platform described above.

In the optical flow algorithm evaluation experiments, all five optical flow algorithms used their respective publicly available pretrained models without fine-tuning on the KITTI Flow 2015 dataset, ensuring evaluation fairness. The specific pretrained models used for each algorithm are listed in Table 1. This part of the experiments was implemented using Python 3.9, CUDA 11.3, cuDNN 8.0, and PyTorch 1.12.0 (PyTorch Foundation, Linux Foundation, San Francisco, CA, USA).

In the comparison experiments with mainstream video action recognition methods, 2 s video clips were extracted for slipping events, and the remaining videos were segmented into clips of equal duration, yielding 31 slipping clips and 288 normal walking clips. These were divided into training, validation, and test sets at a ratio of 7:1.5:1.5. MViT, SlowFast, and STME were initialized with pretrained weights MVIT_B_16x4, SLOWFAST_8x8_R50, and resnet50-19c8e357, respectively, and fine-tuned on this dataset for 50 epochs.

In the cross-farm generalization validation experiments, the 17 single-cow walking videos from Dataset 2 were used. Fifteen frames were uniformly sampled from each cow’s walking video for key point annotation, yielding a dataset of 255 annotated frames, which were divided into training and test sets at a ratio of 8:2. The model was trained for 60,000 iterations, with all other parameter configurations consistent with those described above. In the multi-cow scenario exploration, 15 multi-cow walking videos from Dataset 2 were used, with 30 frames annotated per video, yielding a total of 450 annotated frames. These were divided into training and test sets at a ratio of 8:2. The model backbone used the default DLCRNet architecture with imgaug augmentation and was trained for 150,000 iterations.

## 3. Results

### 3.1. Hoof Key Point Localization Performance

#### 3.1.1. Backbone Network Comparison

**Figure 11:**
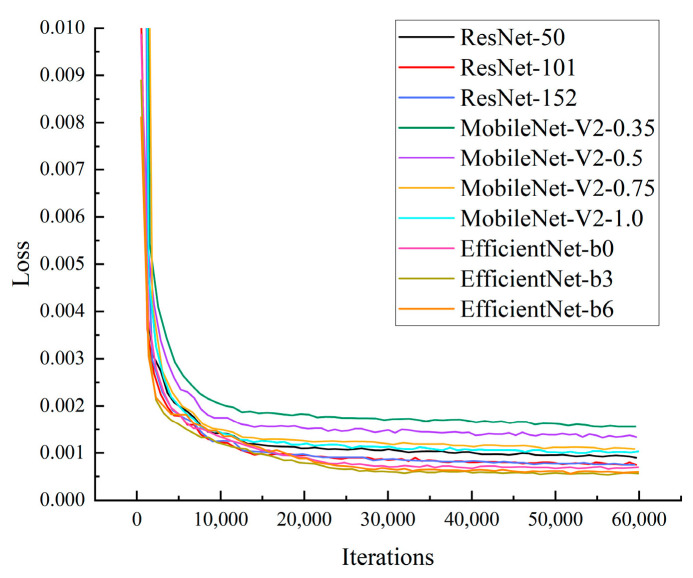
Loss values during the training of different backbone network models.

As shown in Figure 11, all ten backbone networks converged at 30,000 iterations. The networks with the highest loss values, from smallest to largest, were EfficientNet, ResNet, and MobileNet-V2, with EfficientNet-b3 consistently obtaining the lowest loss value. An 11 s video of a cow walking sideways was subsequently used as input to compare and analyze the performances of the candidate backbone networks. The key point positioning error and computational efficiency are presented in Table 2. The MobileNet series exhibited the shortest inference time but had a larger error. ResNet demonstrated a computational speed similar to that of EfficientNet but achieved better accuracy than the other two models. Therefore, ResNet-50 was chosen as the backbone network for optimization. Although the EfficientNet series of networks are small, they perform poorly in terms of inference speed. This may be attributed to the EfficientNet series utilizing a large number of low FLOPs and many data read/write operations, while the GPU’s memory access bandwidth is limited, causing the model to spend considerable time reading and writing data from video memory and failing to fully utilize the GPU’s computational power [38].

#### 3.1.2. CA-Enhanced ResNet-50 Localization Performance

**Figure 12:**
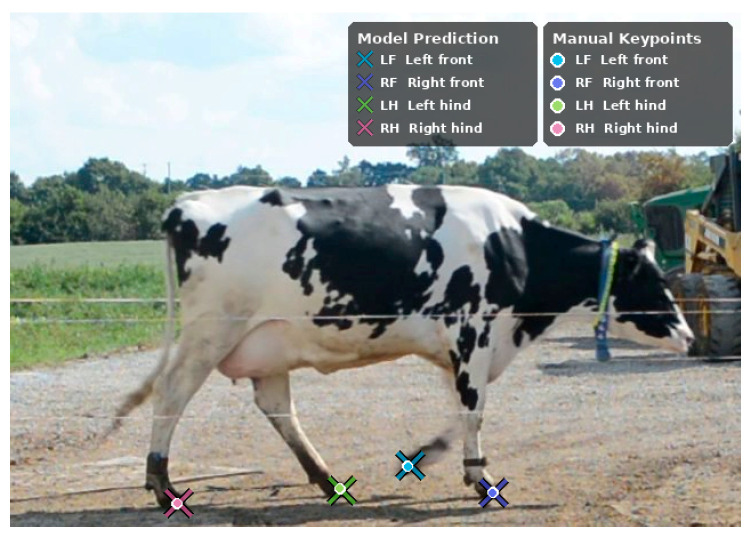
Localization results of the CA-enhanced ResNet-50.

The positioning results of the enhanced ResNet-50 model are presented in Figure 12. As shown in the figure, the enhanced model achieves relatively accurate positioning results, with minimal error compared with manual annotations.

To validate the rationale behind the selection of the CA mechanism, this study designed ablation experiments to systematically compare four attention mechanisms—SE, ECA, CBAM, and CA—under identical integration positions and unified training configurations, with a no-attention baseline included for reference. To ensure statistical reliability, each configuration was independently trained five times with different random seeds, and results are reported as mean ± standard deviation. The results are presented in Table 3. CA achieved the best mean performance on both training and test error metrics, outperforming the second-best CBAM by 0.26 px and 0.22 px in training and test error, respectively, and outperforming the no-attention baseline by 0.41 px and 0.50 px. The performance advantage of CA stems from its lightweight bidirectional spatial encoding mechanism; by establishing positional awareness along the horizontal and vertical directions separately, CA can complete feature recalibration in the channel dimension while preserving spatial structural information. Compared to attention mechanisms that focus solely on the channel dimension, such as SE and ECA, CA achieves effective synergy between channel importance and spatial coordinate information, making it better suited for precise key point localization tasks.

Table 4 compares the test errors of three methods: the CA-enhanced ResNet-50, the original ResNet-50, and YOLOv8s-pose. The results indicate that the CA-enhanced ResNet-50 achieved the lowest test error, reducing the mean test error from 3.30 px to 2.80 px relative to the original ResNet-50, and achieving a 30.9% reduction compared to YOLOv8s-Pose. Overall, these results demonstrate that the incorporation of CA effectively improves the small-target localization capability of the ResNet-50 network.

**Figure 13:**
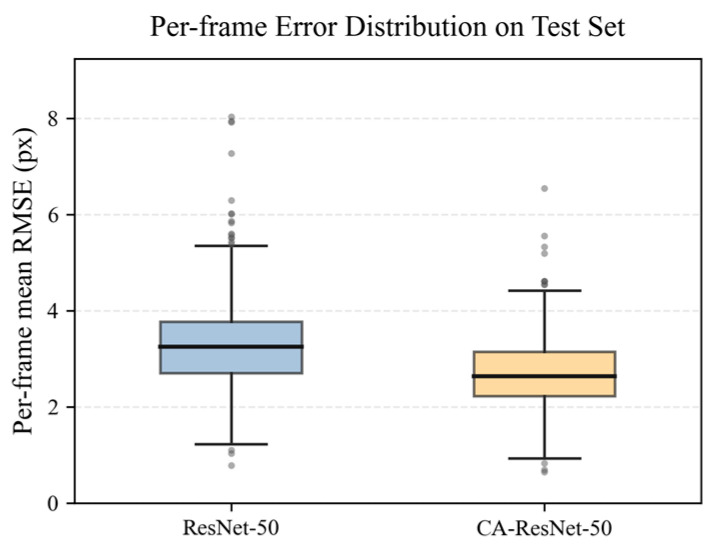
Per-frame error distribution of ResNet-50 and CA-ResNet-50 on the test set.

To further examine the per-frame localization stability, box plots of the per-frame mean RMSE on the test set were constructed for both the baseline ResNet-50 and the CA-enhanced ResNet-50, as shown in Figure 13. The baseline ResNet-50 exhibited a median per-frame RMSE of 3.24 px, whereas the CA-enhanced ResNet-50 reduced the median to 2.64 px, representing an 18.7% reduction. The CA-enhanced model also produced a narrower interquartile range and fewer high-error outlier frames, indicating a more concentrated error distribution across the test set. These results confirm that the CA module not only reduces the overall localization error but also yields more consistent key point predictions across frames.

**Figure 14:**
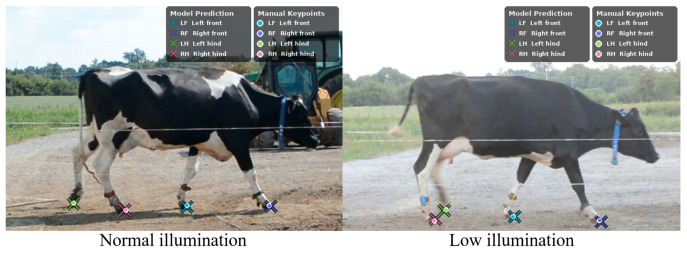
Localization results under different illumination conditions.

Figure 14 presents the key point localization results of the model under two lighting conditions: normal, and low illumination. As shown in the figure, the model achieves relatively accurate localization of cow hoof key points across all lighting conditions, with minimal deviation between predicted points and manual annotations. To further quantitatively evaluate the robustness of the model under different lighting conditions, this study categorized 200 test images into two lighting conditions. The categorization was based on the overall brightness and shadow distribution observed across the cow’s body surface and ground regions: normal illumination refers to conditions with uniform lighting or the presence of obvious direct sunlight (132 images); and low illumination refers to conditions captured on overcast days, where no direct sunlight is present throughout the image (68 images). On this basis, the root mean square errors between the model-predicted key points and manual annotations were calculated separately, with the results presented in Table 5.

As shown in Table 5, the overall RMSE of the model under normal and low-illumination conditions was 2.69 px and 2.93 px, respectively, both remaining below 3 px, with a difference of only 0.24 px. These results indicate that the improved DeepLabCut model maintains stable localization performance across different natural lighting scenarios, demonstrating good robustness to illumination variation.

### 3.2. Optical Flow Algorithm Selection

Table 6 presents the evaluation results of five optical flow algorithms on the KITTI Flow 2015 dataset, with all algorithms tested using pretrained models that exclude KITTI fine-tuning data to ensure evaluation fairness. As shown in Table 6, NeuFlow v2 achieves the best performance across all accuracy metrics in both non-occluded and occluded regions, with an AEE_noc of 2.32 px and AEE_occ of 4.09 px, while maintaining an inference speed of 14.68 FPS. FlowFormer++ achieves comparable accuracy in non-occluded regions but at only approximately 1 FPS, making it unsuitable for continuous video analysis. GMFlow, SEA-RAFT, and FlowNet2.0 all exhibit notably higher error metrics than NeuFlow v2, with FlowNet2.0 ranking last, reflecting the limitations of early deep learning-based optical flow methods in generalizing to real-world scenarios. Taking both accuracy and inference efficiency into consideration, NeuFlow v2 was ultimately selected as the optical flow extraction algorithm for hoof motion analysis.

**Figure 15:**
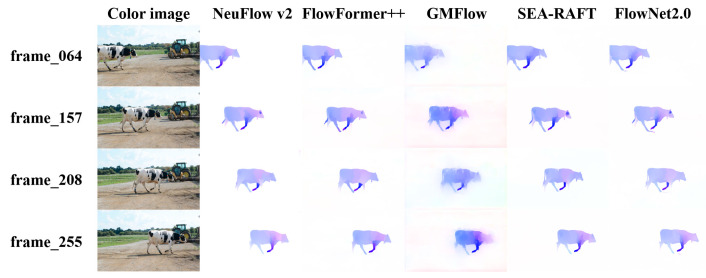
Optical flow prediction results for the side-view video of a cow.

The visualization results of optical flow inference on side-view walking videos are presented in Figure 15. NeuFlow v2 produces clean backgrounds with well-localized hoof responses clearly distinguished from the trunk region, and FlowFormer++ achieves comparable background suppression and hoof localization but is limited by its inference speed. GMFlow exhibits large areas of background noise in multiple frames, demonstrating the weakest background suppression. SEA-RAFT achieves acceptable suppression but with partially missing body contour edges, while FlowNet2.0 lacks effective differentiation between hooves and trunk.

To objectively compare background suppression, a background leakage metric was computed over a fixed rectangle above the cow’s back (rows 30–180, columns 40–1240), a static region whose true flow is zero. Since zero motion appears as white and larger motion as more saturated colour, the mean colour saturation in this region served as a measure of spurious background flow, averaged over 400 randomly sampled frames common to all algorithms. As shown in Table 7, GMFlow yields 0.017, about three times that of the others, indicating the weakest suppression, while NeuFlow v2, FlowFormer++ and SEA-RAFT are all low and comparable. FlowNet2.0 attains the lowest value yet the highest endpoint error in Table 6, showing that low leakage does not imply accurate flow.

### 3.3. Motion Feature Extraction Results for Cow Hooves

#### 3.3.1. Analysis of Cow Hoof Movement Speed and Direction Curves

**Figure 16:**
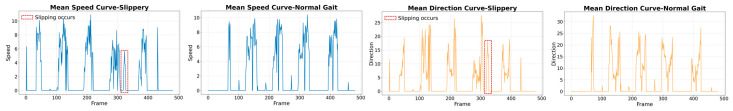
Cow hoof speed curve and hoof motion direction curve.

The speed and direction curves of the cow’s hooves, derived from the optical flow information, are presented in Figure 16. In the speed curve, each normal gait cycle produces a peak corresponding to the hoof’s lifting and landing phases: the curve rises as the hoof begins to move and returns to zero when the hoof contacts the ground and the optical flow vanishes. When slipping occurs, the hoof moves a short distance forward after landing, causing the optical flow to briefly reappear and generating a small abnormal peak following the normal peak.

In the direction curve, the ordinate represents the angle between hoof movement direction and the horizontal plane. During normal walking, the curve exhibits sharp peaks at both ends, corresponding to the upward lifting and downward landing phases, with a concave shape in the middle. When slipping occurs, an abnormally small peak emerges after the normal landing peak, corresponding to the brief forward movement of the hoof.

Therefore, an abnormal peak appears in both the speed and direction curves following the normal peak when slipping occurs. The differences in the horizontal coordinates of adjacent peaks are selected as the slipping feature parameter, within which abnormally low values indicate slipping events.

#### 3.3.2. Extraction Results for the Slipping Feature Parameter

**Figure 17:**
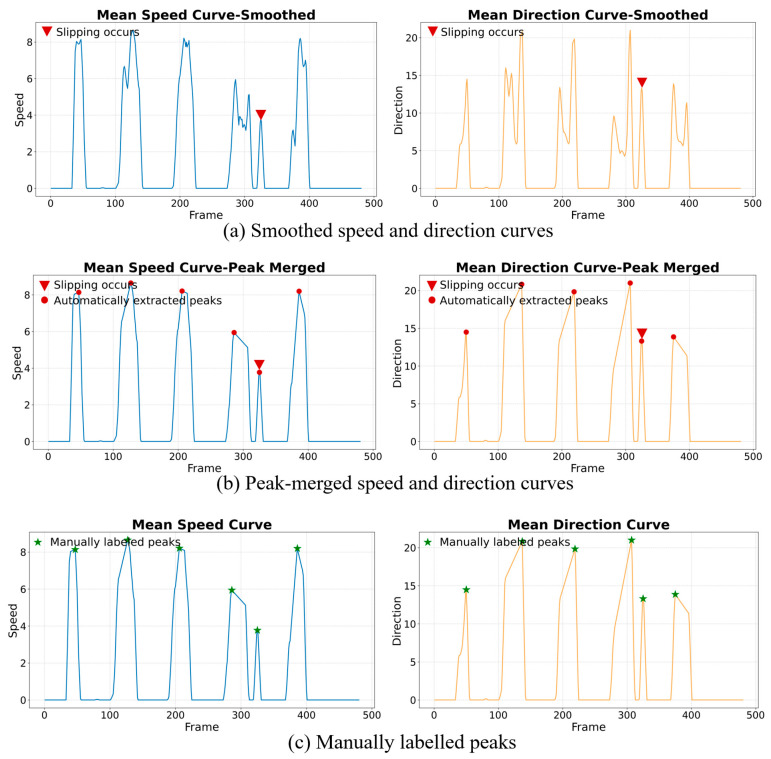
Feature extraction results for cow hoof slipping.

After the speed and direction curves for the cow’s four hooves were obtained, the curves were subjected to two rounds of filtering. The filtered speed and direction curves for the slipping hooves are depicted in Figure 17a, showing a satisfactory smoothing effect. The curves after peak merging and extraction are shown in Figure 17b, where multiple peaks have been effectively consolidated and the moments of slipping are marked accordingly. As described in Section 3.3.1, abnormally low values in the slipping feature array indicate slipping events, as the abnormal peak is very close to the normal peak, resulting in a small difference between the horizontal coordinates of adjacent peaks. The results of manually marking the peak points are shown in Figure 17c; the manually marked peak points exhibit a high degree of overlap with the automatically identified peak points, indicating that the algorithm can accurately extract the peak points after the aforementioned processing.

### 3.4. Slip Detection Performance

#### 3.4.1. Ablation Experiment on Pixel Region Size

To validate the rationale behind the selection of the hoof optical flow extraction region size (20 × 20 pixels), ablation experiments were conducted on three pixel region sizes—15 × 15, 20 × 20, and 30 × 30—to evaluate the classification performance of Random Forest (RF) and XGBoost under each size, and the results are presented in Table 8 and Table 9.

As shown in Table 8 and Table 9, the 20 × 20 pixel region achieved the best overall performance across both feature types. The 15 × 15 region, due to its excessively small receptive field, is susceptible to hoof key point localization errors, resulting in the incomplete capture of hoof motion information. Although the 30 × 30 region has a larger receptive field, it introduces more interfering optical flow during hoof overlap, leading to a decline in performance. The 20 × 20 region effectively covers the hoof motion range while suppressing interfering optical flow, and was adopted for all subsequent experiments.

Regarding classifier selection, RF consistently outperformed XGBoost across both feature types at the 20 × 20 size, achieving the best F1 score of 91.8% and AUC of 0.995 on speed direction features. Therefore, Random Forest was ultimately adopted as the slip classification model.

#### 3.4.2. Slip Classification Results

As shown in Table 8, the Random Forest model based on speed magnitude features achieved an accuracy of 98.3%, precision of 96.0%, recall of 77.4%, F1 score of 85.7%, and AUC of 0.989. As shown in Table 9, the model based on speed direction features achieved an accuracy of 98.9%, precision of 93.3%, recall of 90.3%, F1 score of 91.8%, and AUC of 0.995.

Compared with the speed magnitude model, the direction-feature model achieved higher recall (90.3% vs. 77.4%), indicating more comprehensive coverage of slip events and fewer missed detections. The speed magnitude model achieved slightly higher precision (96.0% vs. 93.3%), reflecting a marginally stronger capability in suppressing false positives. The two feature types thus exhibit a complementary relationship in the precision–recall trade-off. For an early-warning welfare-monitoring tool, however, the two metrics are not equally important. A missed slipping event means that a potential hoof-health problem or hazardous floor condition goes undetected, whereas an occasional false positive only leads to a redundant check. Recall is therefore the more critical metric in this application, and the higher recall of the direction-feature model makes it the more suitable choice for practical deployment. Considering all metrics collectively, the direction-feature model achieved higher accuracy (98.9% vs. 98.3%), F1 score (91.8% vs. 85.7%), and AUC (0.995 vs. 0.989), demonstrating superior overall classification performance. This suggests that changes in hoof movement direction possess stronger biomechanical indicative significance for slip behaviour, enabling more stable and accurate discrimination between slipping and normal gait.

To assess the stability of the direction-feature Random Forest, a further analysis was carried out with 20 repetitions of grouped five-fold cross-validation. The mean F1 score was 0.901 ± 0.078 (95% empirical interval: 0.738–1.000), the mean recall was 0.906 ± 0.113, the mean precision was 0.911 ± 0.108, the mean accuracy was 0.986 ± 0.011, and the mean AUC was 0.996 ± 0.005. These means are close to the single-run values reported in Table 9. The repeated-run statistics are listed in Table 10.

**Figure 18:**
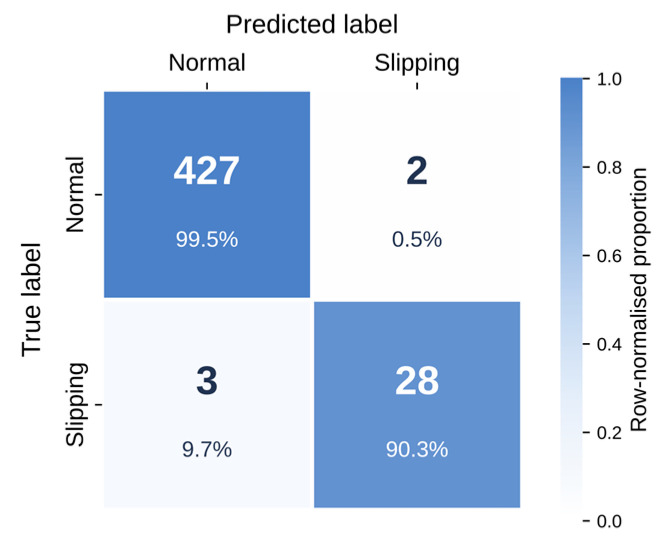
Confusion matrix of the direction-feature Random Forest.

The aggregated confusion matrix is shown in Figure 18. Among the 31 slipping samples, 28 were correctly detected and three were missed. Two of the 429 normal samples were flagged as slipping. The per-fold results in Table 10 show that recall ranged from 0.667 to 1.000 and precision from 0.778 to 1.000 across the five folds. The width of these intervals is attributable to the limited number of abnormal samples in this study.

### 3.5. Slipping Distance Detection Accuracy

Cow hoof slip distances were manually recorded in 31 videos of cow hoof slipping. The start and end times of the cow hoof slipping were determined, and the slip distance was measured in pixels accordingly, as shown in Figure 1, where AA′ represents the slip distance.

Table 11 presents partial manual annotation results and model prediction results. The root mean square error (RMSE) between the manual annotation results and the predicted slipping distance was 1.22 pixels, which corresponds to an actual physical error of approximately 6.48 mm based on a pixel-to-physical conversion factor of 5.31 mm/pixel. The cow hoof-slipping distance typically ranges from 10 to 20 pixels, corresponding to an actual physical distance of approximately 53.1 mm to 106.2 mm, whereas the prediction error is only approximately 6.48 mm, representing approximately 6.1% to 12.2% of the typical slipping distance range, indicating a high degree of agreement between predicted and true values and demonstrating good accuracy in hoof-slipping distance detection.

**Figure 19:**
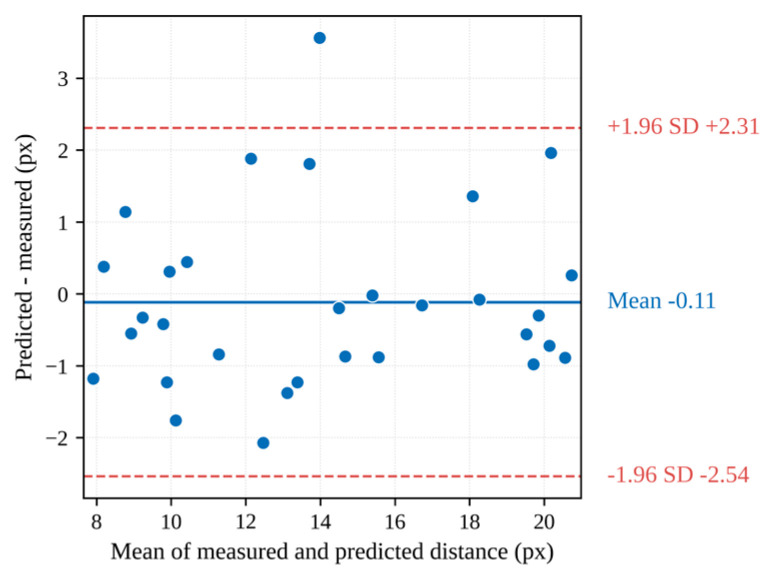
Bland–Altman analysis of the predicted and manually measured slipping distances. The solid line denotes the mean difference and the dashed lines denote the 95% limits of agreement.

To further quantify the distribution of the slipping distance estimation error, a complete analysis was carried out on the manual measurements and predictions for all 31 videos. The mean absolute error was 0.96 px, equivalent to 5.10 mm, with a 95% confidence interval of 0.71 to 1.24 px. The Pearson correlation coefficient between the predicted and manually measured distances was 0.959, with a 95% confidence interval of 0.917 to 0.980. Figure 19 shows the Bland–Altman analysis. The mean difference between the predicted and measured distances was −0.11 px, and the 95% limits of agreement were −2.54 to +2.31 px, equivalent to −13.47 to +12.26 mm. Of the 31 samples, 30 fell within these limits, and the difference did not widen as the slipping distance increased.

## 4. Discussion

### 4.1. Analysis of CA-Enhanced Key Point Localization

In this study, the CA module was incorporated at the end of the residual branch of each Bottleneck block in ResNet-50, prior to the element-wise addition with the identity mapping. This design enables the CA module to function continuously at every depth level, achieving layer-by-layer refinement from low-level texture features to high-level semantic representations and ensuring that spatial positional information is effectively preserved throughout the entire feature extraction process. By establishing positional awareness along the horizontal and vertical directions separately, CA achieves effective synergy between channel importance and spatial coordinate information, outperforming channel-only attention mechanisms such as SE and ECA. The ablation experiment results confirm that CA achieved the lowest mean training and test errors of 2.22 px and 2.80 px, respectively.

Accurate key point localization directly impacts subsequent slip detection. Since optical flow information is extracted within a local 20 × 20 pixel region surrounding each hoof key point, significant localization deviation would shift this region away from the true hoof position, contaminating the extracted optical flow with background motion and impairing the discriminability of slip-related features. The improvement brought by CA ensures that the optical flow extraction region remains consistently anchored within the true hoof area, yielding high-quality motion parameter curves that enhance the accuracy and robustness of the downstream Random Forest classifier.

### 4.2. Advantages of the Proposed Method

#### 4.2.1. Improved Motion Parameter Extraction

Without optical flow, hoof kinematics relied solely on key point trajectories. The cow hoof motion speed curve, derived from key point positioning, is inferred from the curve composed of the key cow hoof points, representing only single-point coordinates and thus capturing motion parameters with limited accuracy. In contrast, optical flow estimates motion by analyzing pixel-level displacements between consecutive frames, yielding average speed and direction information across a local hoof region for each frame and thereby providing a more detailed characterization of hoof movement.

**Figure 20:**
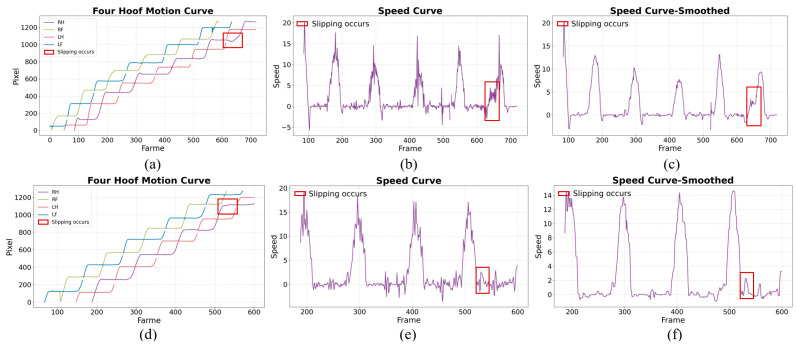
Cow’s four-hoof curves and slipping hoof speed curve. (**a**,**d**) four-hoof horizontal motion curves; (**b**,**e**) speed curves; (**c**,**f**) speed curve-smoothed.

Figure 20a,d shows the four-hoof horizontal motion curves of two cows with right hind-hoof (RH) slipping, with slipping moments marked. In Figure 20a, slipping occurs as the RH moves opposite to the forward direction at movement onset; in Figure 20d, the RH slides forward after landing. The corresponding speed curves are shown in Figure 20b,e, and the filtered speed curves with slipping moments indicated are presented in Figure 20c,f.

**Figure 21:**
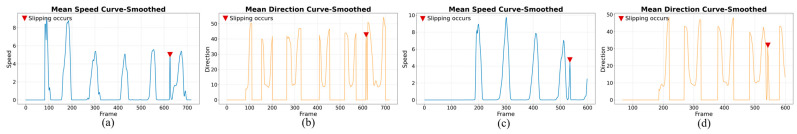
Motion parameter curves for dairy cows. (**a**,**c**) speed curves; (**b**,**d**) direction curves.

The speed and direction curves extracted using the optical flow algorithm are presented in Figure 21. Figure 21a,b shows the RH curves of the cow in Figure 20a, while Figure 21c,d corresponds to the cow in Figure 20d, with slipping moments marked.

As illustrated in Figure 20 and Figure 21, slight hoof slipping is not readily apparent in the four-hoof motion curves, as these curves are derived from single-point key point coordinates that may not fully capture subtle slipping events. By incorporating optical flow information from a 400-pixel region surrounding each hoof, the proposed method provides a more detailed characterization of hoof movement, improving the accuracy of both speed and direction extraction.

#### 4.2.2. Enhanced Robustness

**Figure 22:**
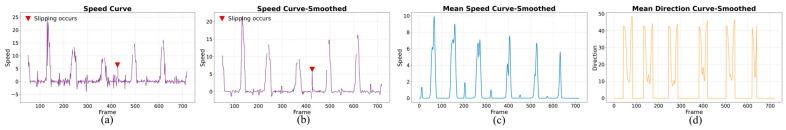
Cow slipping hoof speed curve and motion parameter curve. (**a**) speed curve; (**b**) speed curve-smoothed; (**c**) mean speed curve-smoothed; (**d**) mean direction curve-smoothed.

In the side-view video of a walking cow, the hoof closer to the camera may occlude the hoof farther from the camera. In this study, the cow’s right hooves occluded the left hooves. Figure 22a presents the motion speed curve for the left front hoof (LF) of the cow shown in Figure 20a, and Figure 22b depicts the speed curve after filtering. Figure 22c,d shows the corresponding speed and direction curves extracted using the proposed method.

As depicted in Figure 22a, owing to occlusion, the left front hoof is not recognized in the key point positioning coordinates, resulting in a “breakpoint” in the curve. During subsequent filtering, this “breakpoint” makes it difficult to filter out extraneous peaks, leading to abnormal peaks in the speed curve and affecting the slipping feature, as shown in Figure 22b. After the optical flow algorithm is incorporated, the optical flow image for the “breakpoint” period is not extracted until the left hoof reappears. Although the left hoof is partially occluded at this time, the selection of a 20 × 20 optical flow area ensures that the hoof’s optical flow information is still captured. At the “breakpoint” moment, the overall speed and direction values are set to values greater than threshold *a* for peak merging, thereby mitigating the impact of “breakpoints” on slipping feature extraction. This demonstrates that even when the hoof is occluded, the optical flow algorithm can correctly extract its motion parameters.

**Figure 23:**
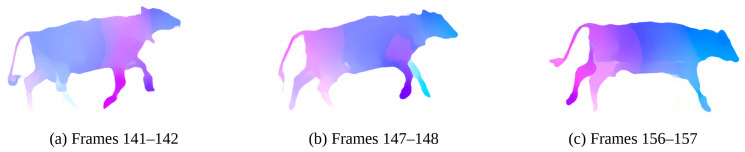
Optical flow prediction results of NeuFlow v2 for a running cow.

Beyond occlusion, rapid hoof movement could introduce motion blur that de-grades optical flow quality. However, Dataset 1 was recorded at 59.94 fps, at which the inter-frame hoof displacement remains small even during the fastest phases of the gait cycle, making motion blur unlikely to occur. To verify this empirically, optical flow was extracted from a video segment of a cow at running pace. As shown in Figure 23, the NeuFlow v2 output remained stable, with no visible degradation in hoof region flow vectors relative to normal walking sequences (Figure 15). These results suggest that motion blur is unlikely to affect the reliability of the proposed framework under the acquisition conditions of this study.

Slipping features are extracted from the optical flow within a 20 × 20 pixel window centred on each predicted hoof key point. Since mean speed and direction are computed only over moving pixels remaining after white-background removal, pixels entering from the window edge under centroid drift are largely discarded, leaving the statistics governed by the same moving pixels as before.

**Figure 24:**
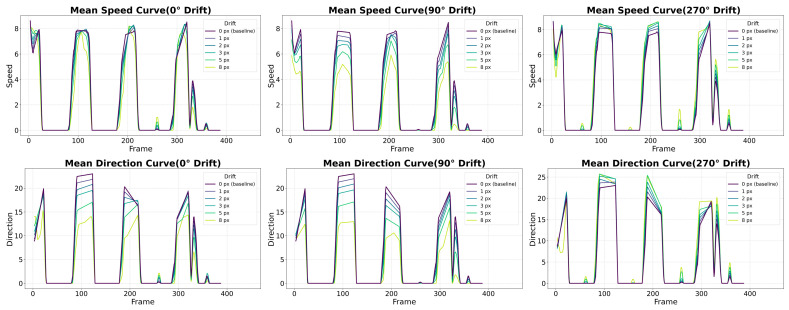
Mean speed and direction curves of the LF hoof under simulated centroid drift.

To verify this robustness, a perturbation test was carried out on the slipping hoof. The window centre was displaced by zero, one, two, three, five and eight pixels in three directions (0°, 90°, 270°), and the slipping interval, defined as the gap between adjacent peak horizontal coordinates, was re-extracted from the full pipeline. Overlaid curves and extracted intervals are shown in Figure 24 and Table 12, respectively. Within the detector’s realistic localisation error, that is for displacements up to three pixels, the direction interval remained at 12 frames (0.20 s at 59.94 fps) in all three directions. The speed interval held at 12 frames up to two pixels, then drifted at three pixels to between 13 and 17 frames in two of the three directions, while staying far below the normal gait interval, so the slip remained detectable. Since the detector’s localisation error is 2.80 pixels, both features keep the slip detectable across the realistic range. At five and eight pixels, which exceed this error, the speed peak often fell below the detection threshold, and the direction interval dropped to 11 frames in one direction and was not detected in one case. As classification relies primarily on the direction interval, which is the more robust feature, the framework is robust to realistic centroid drift.

#### 4.2.3. Comparison with Mainstream Video Action Recognition Methods

To further validate the effectiveness of the proposed method, the cascaded detection framework presented in this study was compared against three mainstream end-to-end video action recognition methods: MViT [39], SlowFast [40], and STME [41]. All three are deep learning-based video classification networks capable of directly accepting raw video clips as input and outputting action category predictions without additional feature engineering. Among them, STME shares a similar design philosophy with STM [42], also based on a 2D CNN framework that simultaneously encodes spatiotemporal and motion features.

All three methods were initialized with publicly available pretrained weights—MViT used MVIT_B_16x4, SlowFast used SLOWFAST_8x8_R50, and STME used resnet50-19c8e357—and were fine-tuned on the dataset of this study for 50 epochs. The comparative results are presented in Table 13.

The experimental results demonstrate that the proposed method outperforms all three comparison methods across accuracy, precision, recall, and F1 score. The three comparison methods perform poorly on the hoof slip detection task because they are designed for conventional video action recognition, with the learning objective of capturing global temporal semantic features from entire video clips. Hoof slipping, however, is a transient and localized motion event with extremely short duration, minimal displacement, and abnormal signals concentrated in only a small number of frames within a very limited hoof region. End-to-end networks are prone to suppressing such weak signals amid the background information of the dominant normal frames, resulting in elevated miss detection rates.

In contrast, the proposed method precisely localizes the hoof region through key point detection, extracts local motion parameter curves via optical flow estimation, and identifies slip-related features from these curves, focusing the detection target on transient motion anomalies in the hoof area. This approach fundamentally circumvents the inherent limitations of end-to-end methods in small-target, sparse-anomaly scenarios, thereby achieving superior detection performance on this task.

### 4.3. Slipping Detection Error Analysis

#### 4.3.1. Analysis of Missed Detections

**Figure 25:**
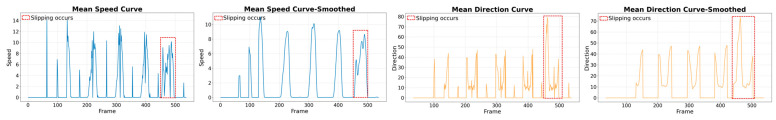
Speed and direction curves for missed hoof detection.

The speed and direction curves for hoof movement in the missed detection cases are shown in Figure 25, with the moment of hoof slipping marked. In the representative run, three of the 31 slipping samples were missed. A case-by-case inspection of their raw motion parameter curves showed that the misses arose from two distinct causes.

The first case was caused by optical flow disappearance. After the hoof slipped and contacted the ground, the optical flow signal weakened and disappeared within a very short time window. This brevity resulted in very few zero values in the motion parameter curve at the moment of hoof slipping, so no distinguishable abnormal peak was formed. The anomalous signal is lost at this stage and cannot be recovered by later processing, leading to a missed detection. This case can be mitigated by raising the acquisition frame rate or by using an optical flow method with stronger temporal resolution.

The other two cases were caused by peak merging. For these samples, a distinguishable abnormal peak is present near the moment of slipping, but it lies too close to the adjacent normal peak, and the curve values between the two peaks remain above the merging threshold a. In the subsequent smoothing process, the normal and abnormal peaks merge, widening the peak of the motion parameter curve at the moment of hoof slipping, so the horizontal coordinate difference between adjacent peaks no longer appears abnormally low. Peak merging of this kind can be mitigated by adjusting the merging threshold a, the prominence parameter, or the smoothing window size, or by adopting a finer peak separation method.

#### 4.3.2. False Positive Analysis

**Figure 26:**
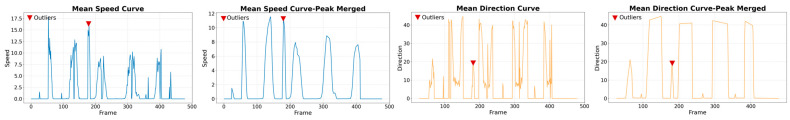
Speed and direction curves for false hoof detection.

Figure 26 shows the speed and direction curves for the hooves of cattle that were misjudged as experiencing slipping during normal walking, with the misjudgement peak marked. Analysis of the key point positioning information and optical flow images revealed that during movement, the left and right hooves briefly crossed and overlapped in terms of key point positioning of hooves. When the left and right or front and back hooves are too close to each other, the positioning of the key points of hooves becomes abnormal. This causes the optical flow image of the hooves, which should have overlap to only a very small degree, to appear as a complete optical flow image of the overlapping hooves, resulting in a peak in the motion parameter curve that is very close to the normal peak. Even if the peak is extracted later by significance, it cannot be filtered out. This leads to normal walking cattle being misidentified as having experienced slipping.

#### 4.3.3. Slipping Distance Error Decomposition

Section 3.5 reports a slipping distance estimation RMSE of 1.22 px (about 6.48 mm). This section decomposes that error to explore the stage from which it originates. As described in Section 2.5.4, the slipping distance is determined only by the X trajectory from key point localisation and by the slipping start and end frames, which are fixed by the peaks of the direction curve. The optical flow does not enter the distance value directly. Because no optical flow ground truth exists for real cow videos, this section substitutes ground truth values for only the two stages for which ground truth is available, namely the key point trajectory and the start and end frames. Replacing each in turn yields four configurations, and the results are listed in Table 14.

When both the key points and the start and end frames are set to ground truth, the residual RMSE is 0.95 px. This residual error is independent of the algorithm. It arises from manual frame reading and pixel-level manual measurement, and reflects the inherent annotation error in this task. Above this baseline, key point localisation alone introduces an error of about 0.47 px. Optical flow and peak detection cannot be separated because the optical flow has no ground truth; together they contribute about 0.59 px.

The distance estimation error therefore contains both a contribution from the algorithm stages and an annotation error that is independent of the algorithm. The latter, at 0.95 px, is the largest component of the total error. In addition, the key point localisation model has an RMSE of 2.80 px, while its contribution to the distance error is only about 0.47 px. This is because the slipping distance is taken as the difference between the X coordinates of the start and end points, and the correlated drift in key point localisation is partly offset through subtraction.

### 4.4. Cross-Farm Generalization Validation

#### 4.4.1. Validation on an Independent Farm Dataset

This section uses Dataset 2 described in Section 2.1 for cross-farm generalization validation. To provide a preliminary assessment of the transferability of the proposed method across different farm environments, a cross-farm validation experiment was conducted at Shengsheng Farm in Luoyang, Henan Province, China.

Compared with the original farm, the new farm differs significantly in floor material, recording equipment, capture parameters, and geographical location, with a time interval of approximately 10 years between the two data collection periods. These differences provide an effective means of examining the proposed method’s adaptability to varying hardware conditions, ground surface environments, and farm management practices.

**Figure 27:**
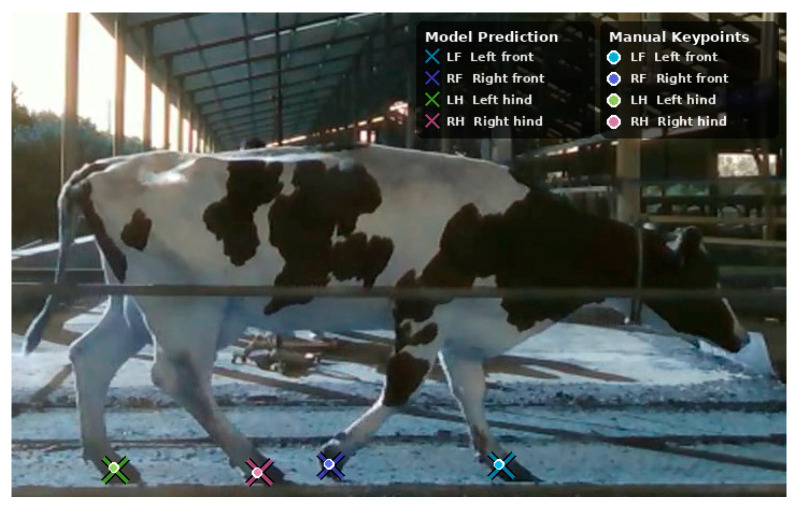
Four-hoof key point localization results on new farm dairy cow data.

For hoof key point localization, the CA-enhanced DeepLabCut model was retrained using data from the new farm. Because the model was retrained on Dataset 2 rather than applied directly to it, this experiment evaluates the adaptability of the framework after retraining on a new environment, rather than the direct generalization of the Dataset 1 model to Dataset 2. The key point localization results on the new farm data are presented in Table 15, and the visualization of localization errors is shown in Figure 27.

**Figure 28:**
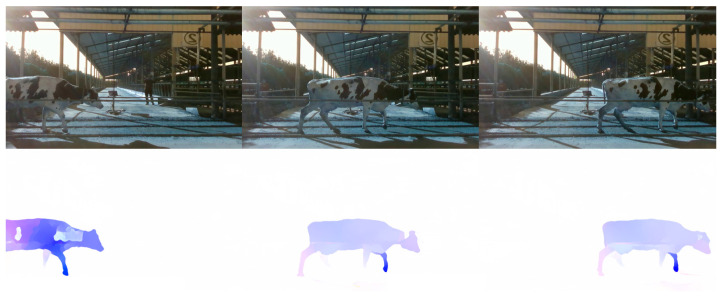
Optical flow inference results on new farm dairy cow data.

As shown in Table 15, the test error of 3.90 px on the new farm at a p-cutoff of 0.6 is slightly higher than the 2.80 px on the original farm. This rise may stem from two factors: the cross-farm domain difference (camera model, floor material, capture parameters) and the smaller amount of annotated data on the new farm. To identify the main contributing factor, a CA-ResNet-50 model was retrained on the original farm data with the number of annotated frames limited to 255, matching the new farm, while all other training and split settings were kept unchanged. At a p-cutoff of 0.6, this model gave a test error of 3.63 px. Training on the full 1000 frames gave 2.80 px; reducing the training scale alone to 255 frames therefore accounts for an increase of approximately 0.83 px. Under the same training scale of 255 frames, the gap between the new farm and the original farm was only 0.27 px, which reflects the effect of the domain difference. The elevated test error on the new farm therefore arises mainly from the limited scale of annotated data, while the residual effect of the domain difference is small. Nevertheless, the localization accuracy of 3.90 px remains sufficient to meet the requirements of subsequent optical flow feature extraction. NeuFlow v2 was then applied to perform optical flow inference on the new farm data, with partial results presented in Figure 28.

For slip feature extraction, a 20 × 20 pixel region was consistently adopted to extract both speed magnitude and direction features. For slip detection, a total of 68 samples were collected, comprising 58 normal samples and 10 abnormal samples. The abnormal samples originated from six cows exhibiting hoof-slipping behaviour, with certain individuals showing slipping in multiple hooves. Five-fold cross-validation was employed for evaluation, and the classification results are presented in Table 16.

As shown in Table 16, the Random Forest classifier based on speed direction features achieved an accuracy of 95.6%, precision of 81.8%, recall of 90.0%, F1 score of 85.7%, and AUC of 0.991 on the new farm data. The classifier based on speed magnitude features achieved an accuracy of 94.1%, precision of 80.0%, recall of 80.0%, F1 score of 80.0%, and AUC of 0.979. Both feature types yielded good detection performance, remaining at a comparable level to the results obtained on the original farm dataset, where the direction-feature model achieved an F1 score of 91.8% and AUC of 0.995. However, this evaluation is based on a small second-farm dataset of only 68 hoof-level samples, of which 10 are abnormal. In addition, the localisation model was retrained on the new farm annotated frames rather than transferred directly. These results therefore provide preliminary evidence of transferability across different farm environments, floor surface types, and recording conditions, rather than definitive proof of generalization. Confirming generalization would require larger-scale validation on fully independent data.

#### 4.4.2. Preliminary Exploration of Multi-Cow Complex Scenarios

This section uses Dataset 2 described in Section 2.1 to explore multi-cow complex scenarios. As all current detection data were collected using a single-cow, side-view setup, complex scenarios involving multiple cows walking simultaneously in real farm environments have not yet been addressed. To investigate this challenge, an additional 15 videos of multiple cows walking in parallel were collected at the new farm, with 30 frames annotated per video, yielding a total of 450 frames. The hoof key point localization results in multi-cow scenarios are presented in Table 17. The RMSE was 4.89 px on the training set and 8.30 px on the test set. After applying a p-cutoff of 0.6, the training error was 4.80 px and the test error decreased to 6.96 px. This suggests that the model’s confidence estimates are well-aligned with actual localization errors: low-confidence predictions consistently correspond to larger localization deviations. The p-cutoff filtering therefore serves as a practical mechanism for improving detection reliability.

**Figure 29:**
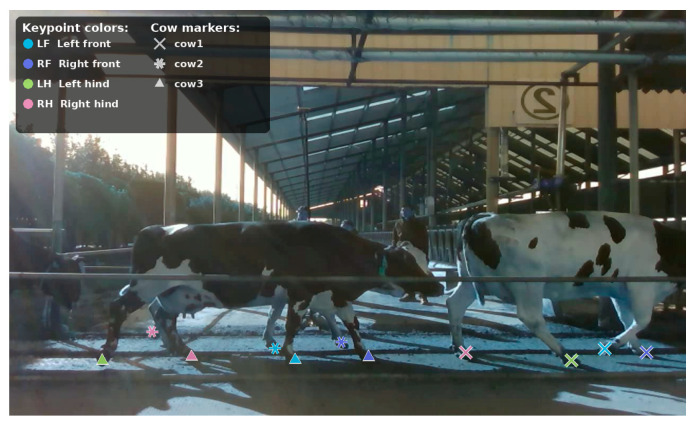
Key point localization results on multi-cow data from the new farm.

To further evaluate the model’s stability under varying levels of scene complexity, the 90-frame test set was grouped according to the number of cows simultaneously present in each frame, where n denotes the number of test frames in each group. As shown in Table 18, as the number of cows in the frame increased from one to four or more, the mean RMSE ranged from 5.95 to 7.30 px, with a variation of only 1.35 px. The standard deviation across groups remained at comparable levels, ranging from 1.98 to 2.33 px. These results indicate that an increasing number of cows in the frame did not notably affect key point localization accuracy. The experimental results demonstrate that the framework is capable of accurately distinguishing individual dairy cows and simultaneously tracking hoof key points across multiple animals. Partial localization results are presented in Figure 29.

These results concern hoof key point localization only. Slip classification under multi-cow conditions has not yet been experimentally evaluated, as no multi-cow slipping samples were collected in this study. Based on the individual identification capability demonstrated above, the slip detection pipeline could in principle be extended to multi-cow scenarios. This would involve extracting speed features from the hoof key points of each cow separately. This extension remains hypothetical at present and requires dedicated validation on multi-cow slipping data. A further challenge is that when the hooves of one cow are completely occluded by an adjacent cow, speed features cannot be extracted owing to the absence of valid image information.

### 4.5. Limitations

Several limitations should be acknowledged. First, the sample size is small. Dataset 1 yielded 460 hoof-level samples, of which 31 were slipping cases. The second-farm dataset contained only 68 hoof-level samples, of which 10 were slipping cases. The limited number of slipping events constrains the statistical power of the evaluation and increases the uncertainty of the reported metrics.

Second, the data are severely class-imbalanced, with a ratio of 31 abnormal to 429 normal samples in Dataset 1. To mitigate the resulting bias toward the majority class, the Random Forest was trained with the class_weight parameter set to “balanced,” and recall for the slipping class was adopted as a primary metric. The class imbalance nonetheless remains a limitation, as the minority class is represented by few examples.

Third, the hoof samples may not be fully independent. The four hooves of one cow share individual-level factors such as gait rhythm, illumination, and viewpoint. The same cow may also appear in more than one video. Grouped cross-validation at the video level was used to prevent leakage within a single recording, but residual non-independence across recordings of the same cow cannot be entirely excluded.

Fourth, the comparison with mainstream action recognition methods has limitations. Owing to the constraints of our data, the comparison used the original input forms of these methods, without additionally introducing ROI cropping or pose-guided enhancement. Both of these enhancements would first require hoof localisation or pose information, which is consistent with the design rationale of this study, namely anchoring the hoof region first and then analyzing motion within that region. We therefore retained the original comparison setting, as it presents the difference in design rationale between end-to-end methods and our method more clearly. We note, however, that this comparison did not incorporate the above enhancements, which is a limitation of the current evaluation.

In addition, the proposed method was evaluated retrospectively on offline datasets and has not yet been validated in a prospective real-time deployment setting. Future studies should further assess its robustness and practicality under real farm-operating conditions. Furthermore, although the proposed method demonstrated promising performance in slipping detection, its practical value would be strengthened by future validation against clinical hoof-health outcomes and objective measurements of floor slipperiness.

### 4.6. Future Research Directions

This study proposes a hoof slip extraction and detection method for dairy cows that combines four-hoof key point localization with optical flow estimation, achieving good detection performance in single-cow side-view scenarios. Several directions merit future investigation. First, extending the framework to multi-cow complex scenarios requires addressing the challenge of feature extraction under complete hoof occlusion, potentially through occlusion prediction, multi-view fusion, or trajectory interpolation. Second, the current independent classification based on speed magnitude and direction features may benefit from multi-feature fusion strategies to fully exploit the complementary information from both feature types, and novel backbone architectures could further improve key point localization accuracy. Future research will further expand the scale of cross-farm validation data and conduct more comprehensive generalization evaluations under varying shooting distances, viewing angles, and more complex real-world scenarios, with the goal of improving the robustness of the proposed method across diverse practical environments.

## 5. Conclusions

This study proposed a cascaded detection framework based on improved DeepLabCut and NeuFlow v2 for automated hoof-slipping detection in dairy cows. Incorporating the Coordinate Attention mechanism into the ResNet-50 backbone reduced mean hoof key point localization error to 2.80 px across five independent training runs, which is a 15.2% improvement over the baseline. Based on pixel-level fusion of NeuFlow v2 optical flow with key point coordinates, speed and direction curves were extracted from hoof regions, and slipping features were constructed from horizontal coordinate differences between adjacent motion peaks. The Random Forest classifier achieved an F1 score of 91.8% and an AUC of 0.995, outperforming mainstream end-to-end video action recognition methods.

Cross-farm validation provided preliminary evidence of transferability across different floor surface types, recording equipment, and geographical locations, although it involved retraining on the second farm and a limited number of samples. These results indicate promising feasibility under controlled side-view recording conditions, rather than readiness for broad practical deployment. The framework nonetheless offers a non-invasive basis for early hoof-health monitoring and welfare-oriented farm management in dairy cows. Future work could explore real-time deployment and extension to other hoof-related behavioural indicators to further support automated welfare-monitoring.

## Figures and Tables

**Table 1:** Pretrained models used by different optical flow algorithms.

Optical Flow Algorithm	Pretrained Model
NeuFlow v2	things
FlowFormer++	Chairs, things, things_288960
GMFlow	chairs, things
SEA-RAFT	Tartan-C-T-432 × 960-M
FlowNet2.0	FlowNet2_checkpoint

**Table 2:** Comparison of the performances of different backbone network models.

Networks	Training Error (Pixels)	Test Error (Pixels)	Model Size (MB)	FPS
ResNet-50	2.69	3.31	98	35.72
ResNet-101	2.9	3.61	170.5	27.71
ResNet-152	2.73	3.35	230.6	20.57
MobileNet-V2-0.35	4.73	5.01	4.5	48.0
MobileNet-V2-0.5	4.15	4.34	15.6	48.0
MobileNet-V2-0.75	4.04	4.61	26.5	40.0
MobileNet-V2-1.0	4.0	4.46	52.3	36.0
EfficientNet-b0	3.0	3.53	19.5	33.19
EfficientNet-b3	3.65	4.03	47.08	23.22
EfficientNet-b6	5.78	5.98	165.4	19.43

**Table 3:** Ablation experiment results of attention mechanisms. Bold indicates the best result.

Attention Module	Training Error (Pixels)	Test Error (Pixels)
None (baseline)	2.63 ± 0.07	3.30 ± 0.09
SE	2.59 ± 0.11	3.22 ± 0.08
ECA	2.59 ± 0.11	3.18 ± 0.07
CBAM	2.48 ± 0.09	3.02 ± 0.07
CA (finally adopted)	**2.22 ± 0.09**	**2.80 ± 0.20**

**Table 4:** Comparison of key point localization results across methods. Bold indicates the best result.

Method	Test Error RMSE (Pixels)
ResNet-50 (Baseline)	3.30
YOLOv8s-Pose	4.05
CA-Enhanced ResNet-50 (Ours)	**2.80**

**Table 5:** Key point localization RMSE under different lighting conditions.

Lighting Condition	Sample Size	Overall RMSE (Pixels)
Normal illumination	132	2.69
Low illumination	68	2.93

**Table 6:** Performance metrics of different optical flow algorithms on the KITTI Flow 2015 dataset. Bold indicates the best result.

Algorithm	Pretrained Model	AEE_occ (px)	Fl_occ (%)	AEE_noc (px)	Fl_noc (%)	FPS
NeuFlow v2	things	**4.09**	**14.50**	**2.32**	9.17	14.68
FlowFormer++	chairs	10.94	35.89	6.31	28.58	0.98
FlowFormer++	things	4.54	15.27	2.40	8.80	1.00
FlowFormer++	things_288960	4.48	14.78	2.40	**8.56**	1.00
GMFlow	Chairs	14.21	38.07	7.60	31.35	5.70
GMFlow	things	7.82	23.39	3.35	15.22	5.68
SEA-RAFT	Tartan-C-T-432 × 960-M	6.60	24.50	3.52	17.32	15.94
FlowNet2.0	FlowNet2_checkpoint	11.63	31.96	5.61	24.38	**18.41**

**Table 7:** Mean background saturation of different optical flow algorithms.

Algorithm	Mean Background Saturation
NeuFlow v2	0.0064
FlowFormer++	0.0056
GMFlow	0.0170
SEA-RAFT	0.0068
FlowNet2.0	0.0042

**Table 8:** Speed magnitude features.

Region Size	Model	Accuracy	Precision (Slip)	Recall (Slip)	F1 (Slip)	AUC
15 × 15	RF	97.4%	91.3%	67.7%	77.8%	0.940
15 × 15	XGBoost	98.0%	100.0%	71.0%	83.0%	0.911
20 × 20	RF	98.3%	96.0%	77.4%	85.7%	0.989
20 × 20	XGBoost	97.6%	88.5%	74.2%	80.7%	0.984
30 × 30	RF	97.6%	91.7%	71.0%	80.0%	0.978
30 × 30	XGBoost	97.8%	95.7%	71.0%	81.5%	0.964

**Table 9:** Speed direction features.

Region Size	Model	Accuracy	Precision (Slip)	Recall (Slip)	F1 (Slip)	AUC
15 × 15	RF	98.0%	86.7%	83.9%	85.2%	0.967
15 × 15	XGBoost	97.6%	85.7%	77.4%	81.4%	0.970
20 × 20	RF	98.9%	93.3%	90.3%	91.8%	0.995
20 × 20	XGBoost	98.3%	89.7%	83.9%	86.7%	0.961
30 × 30	RF	98.7%	96.3%	83.9%	89.7%	0.994
30 × 30	XGBoost	98.3%	87.1%	87.1%	87.1%	0.977

**Table 10:** Per-fold and repeated grouped five-fold cross-validation of the direction-feature Random Forest. Folds 1 to 5 correspond to one representative run. The last two rows summarize 20 repeated runs.

Fold/Statistic	Accuracy	Precision (Slip)	Recall (Slip)	F1 (Slip)	AUC
Fold 1	0.978	0.778	1.000	0.875	0.995
Fold 2	0.978	1.000	0.667	0.800	0.998
Fold 3	1.000	1.000	1.000	1.000	1.000
Fold 4	0.989	1.000	0.833	0.909	0.996
Fold 5	1.000	1.000	1.000	1.000	1.000
Mean ± SD	0.986 ± 0.011	0.911 ± 0.108	0.906 ± 0.113	0.901 ± 0.078	0.996 ± 0.005
95% interval	[0.967, 1.000]	[0.667, 1.000]	[0.645, 1.000]	[0.738, 1.000]	[0.983, 1.000]

**Table 11:** Partial manually labelled distance and calculated distance.

Index	1	2	3	4	5	6	7	8	9	10
Manually recorded distance	16.80	18.30	9.80	13.50	11.70	19.20	14.60	8.50	20.20	12.80
Calculated distance	16.64	18.22	10.11	11.43	10.86	21.16	14.40	7.32	19.22	14.61

**Table 12:** Slipping interval features of the LF hoof under simulated centroid drift (frames). Bold values denote the slipping interval; n.d. indicates that the slip peak was not detected.

Drift	Speed 0°	Direction 0°	Speed 90°	Direction 90°	Speed 270°	Direction 270°
0 px	[115, 107, **12**]	[101, 66, 131, **12**]	[117, 107, **12**]	[101, 66, 131, **12**]	[117, 107, **12**]	[101, 66, 131, **12**]
1 px	[97, 107, **12**]	[100, 67, 131, **12**]	[116, 107, **12**]	[101, 66, 131, **12**]	[75, 119, 105, **12**]	[101, 66, 131, **12**]
2 px	[96, 108, **12**]	[100, 67, 131, **12**]	[107, 116, **12**]	[101, 66, 131, **12**]	[77, 117, 105, **12**]	[69, 98, 131, **12**]
3 px	[105, 103, **17**]	[100, 94, 104, **12**]	[98, 115, **13**]	[101, 66, 131, **12**]	[76, 118, 105, **12**]	[69, 98, 131, **12**]
5 px	[104, 104, **17**]	[101, 94, 104, **12**]	[98, 114] (n.d.)	[100, 67, 131, **12**]	[76, 119, 105, **12**]	[69, 98, 132, **11**]
8 px	[97, 108] (n.d.)	[99, 96, 104, **12**]	[98, 114] (n.d.)	[83, 115] (n.d.)	[75, 120, 105, **12**]	[68, 98, 133, **11**]

**Table 13:** Performance comparison with mainstream video action recognition methods.

Method	Accuracy	Precision (Slip)	Recall (Slip)	F1 (Slip)
SlowFast	88.0%	50.0%	50.0%	50.0%
MViT	90.0%	57.1%	66.7%	61.5%
STME	90.0%	60.00%	50.00%	54.55%
Ours (Direction Features + RF)	98.9%	93.3%	90.3%	91.8%

**Table 14:** Error source decomposition of the slipping distance estimation.

Configuration	Residual Error Sources	RMSE (px)	Physical Error (mm)
Fully automatic	Key points + flow + peak + baseline	1.22	6.48
GT key points + auto frames	Flow + peak detection	1.12	5.95
Auto key points + GT frames	Key point localisation	1.06	5.63
GT key points + GT frames	Annotation baseline	0.95	5.04

**Table 15:** Key point localization results of the CA-enhanced DeepLabCut model on the new farm dataset.

Index	Train Error	Test Error	Train Error with p-Cutoff	Test Error with p-Cutoff
Localization Error (RMSE)	2.41	4.01	2.41	3.90

**Table 16:** Slip detection results on new farm data.

Feature Type	Model	Accuracy	Precision (Slip)	Recall (Slip)	F1 (Slip)	AUC
Speed magnitude features	RF	94.1%	80.0%	80.0%	80.0%	0.979
Speed direction features	RF	95.6%	81.8%	90.0%	85.7%	0.991

**Table 17:** Hoof key point localization results of the DeepLabCut model in multi-cow scenarios.

Index	Train Error	Test Error	Train Error with p-Cutoff	Test Error with p-Cutoff
Localization Error (RMSE)	4.89	8.30	4.80	6.96

**Table 18:** Relationship between the number of cows and test set RMSE (p-cutoff = 0.6).

Cows in Frame	n	Test Error	Std
1	16	6.99	2.16
2	40	7.30	2.33
3	27	6.71	2.01
≥4	7	5.95	1.98

## Data Availability

Restrictions apply to the availability of these data. The data were obtained from the University of Kentucky Coldstream Research Dairy Farm and Luoyang Shengsheng Animal Husbandry Co., Ltd. and are available from the corresponding author with the permission of these institutions.
